# The Sexual Goals of Metoidioplasty Patients and Their Attitudes Toward Using PDE5 Inhibitors and Intracavernosal Injections as Erectile Aids

**DOI:** 10.1016/j.esxm.2022.100505

**Published:** 2022-04-08

**Authors:** Amir Khorrami, Sahil Kumar, Elise Bertin, Richard Wassersug, Cormac O'Dwyer, Smita Mukherjee, Luke Witherspoon, Peter Mankowski, Krista Genoway, Alex G. Kavanagh

**Affiliations:** 1 Faculty of Medicine, University of British Columbia, Vancouver, British Columbia, Canada; 2 Gender Surgery Program, Vancouver Coastal Health, Vancouver, British Columbia, Canada; 3 Division of Plastic and Reconstructive Surgery, University of British Columbia, Vancouver, British Columbia, Canada; 4 Department of Urologic Sciences, University of British Columbia, Vancouver, British Columbia, Canada

**Keywords:** Gender-affirming surgery, Metoidioplasty, Phalloplasty, Sexual dysfunction, Erectile dysfunction, Erectile Aids, Phosphodiesterase inhibitors, Intracavernosal injections

## Abstract

**Background:**

Following metoidioplasty, transmen (TM) experience sexual function challenges including erectile dysfunction, which is typically treated in cisgender men with phosphodiesterase-5 inhibitors (PDE5i) and intracavernosal injections (ICI).

**Aim:**

We aim to evaluate sexual function post-metoidioplasty and explore attitudes toward using PDE5i and ICI as potential erectile aids

**Methods:**

All patients who had metoidioplasty completed at the Gender Surgery Program in Vancouver, British Columbia were contacted. Participants completed an electronically accessible self-constructed questionnaire consisting of 39 items on erectile function, orgasm, and penetrative intercourse which also captured Erection Hardness Scores (EHS). Data were analyzed via *t*-test and 1-way ANOVA.

**Outcomes:**

Our outcomes were the importance of erectile function, ability to orgasm, penetrative intercourse, and attitudes towards using PDE5i and ICI post-metoidioplasty.

**Results:**

Fifteen out of 22 patients completed the survey (median age 32 years). Most had metoidioplasty within the past 2 years. The participants ranked the ability to orgasm and to achieve or maintain erections significantly higher than penetrative intercourse (*P* <.001, *P* =.005 respectively). Most participants reported facing challenges with penetrative intercourse (87%) and erectile function (80%). In contrast, a smaller proportion reported challenges with orgasm (33%). With regards to EHS, 83% of participants described their erections as either “larger but not hard,” or “hard but not hard enough for penetration.” A total of 47% of the participants had previously tried PDE5i, but none had used ICI. Although 87% were willing to use PDE5i, only 40% were willing to try ICI to improve their erections. Patients reported lack of knowledge and understanding among primary care physicians as barriers to accessing treatment for sexual dysfunction.

**Clinical Translation:**

The results of this study can facilitate decision making for TM undergoing genital gender-affirmation surgery and provide potential options for improving erectile function post surgery.

**Strengths & Limitations:**

This study represents the first assessment of sexual function and use of erectile aids in post-metoidioplasty patients. The results of this study are limited by the small sample size and enrolment from a single surgical center.

**Conclusion:**

Metoidioplasty patients surveyed fail to achieve a fully rigid erection without treatment, typically retain the ability to orgasm, and are generally willing to try PDE5i.

**Khorrami A, Kumar S, Bertin E, et al. The Sexual Goals of Metoidioplasty Patients and Their Attitudes Toward Using PDE5 Inhibitors and Intracavernosal Injections as Erectile Aids. Sex Med 2022;10:100505.**

## INTRODUCTION

Gender-affirming surgery is broadly categorized into top and bottom (lower) procedures. The three main reported goals of bottom surgeries for transmen (TM) are to confirm their gender and sexual identity, to facilitate voiding while standing, and to enable insertive sexual intercourse.^1^

The two most common lower surgeries performed for TM are phalloplasty and metoidioplasty.^2^ Phalloplasty is undertaken with the goal of producing a neophallus similar in length and girth to that of cisgender men (ie, individual whose gender identity is congruent with their sex assigned at birth) penis, and one capable of erection and penetrative intercourse.^3^

Metoidioplasty is a reconstructive procedure that includes creation of a neophallus from a hormonally hypertrophied clitoris. The clitoral suspensory ligaments and ventral chordee are released to achieve maximal clitoral length prior to urethral reconstruction. The native urethra is lengthened to reach the tip of the glans, allowing voiding while standing, while the scrotum is created from labia majora with two inserted testicular prostheses.^4^ Several studies have reported significant advantages of metoidioplasty over phalloplasty. These include preserved intrinsic erogenous sensation and erection, absence of a need for preoperative hair removal, lack of donor-site morbidity and scarring, decreased operating room times, shorter hospital stays, and lower healthcare costs.^5^, ^6^, ^7^

One limitation of metoidioplasty is that the shorter neophallus is often inadequate for penetrative intercourse. Moreover, there are reports of inability to achieve rigid erections.^8^ A recent study reported more sexual dysfunction after metoidioplasty compared to a TM group that had no genital surgery. Some of the dysfunctions associated with metoidioplasty include difficulties achieving sexual arousal, orgasmic dysfunction, and subsequent fear of sexual contacts.^9^ This may be due to dissatisfaction with the aesthetic outcome, or the inability to achieve rigid erections sufficient for penetration.^10^ Ultimately, there is a lack of data and more qualitative information on post-operative sexual outcomes is needed.

Erectile aids are widely used by cisgender men to treat sexual dysfunctions. Phosphodiesterase 5 inhibitors (PDE5i) are the first-line medical therapy for erectile dysfunction (ED). Intracavernosal injections (ICI) of vasodilatory agents such as alprostadil and papaverine are also highly efficacious for ED as a second-line treatment for patients who do not respond well to PDE5i.^11^ Physiologically, PDE5i enhance the vasodilatory effect of nitric oxide, resulting in smooth muscle relaxation and inflow of blood to the corpus cavernosum (erectile tissue) of the penis. The clitoral shaft consists of two parallel erectile corpora cavernosa tissue, Therefore, it is subject to similar physiological effects in response to PDE5i. Canadian, American, and European urology guidelines for sexual dysfunction offer no recommendations for treating ED in metoidioplasty patients.^12^^,^^13^ Furthermore, there is no clear definition of ED in this population.

**Figure 1:**
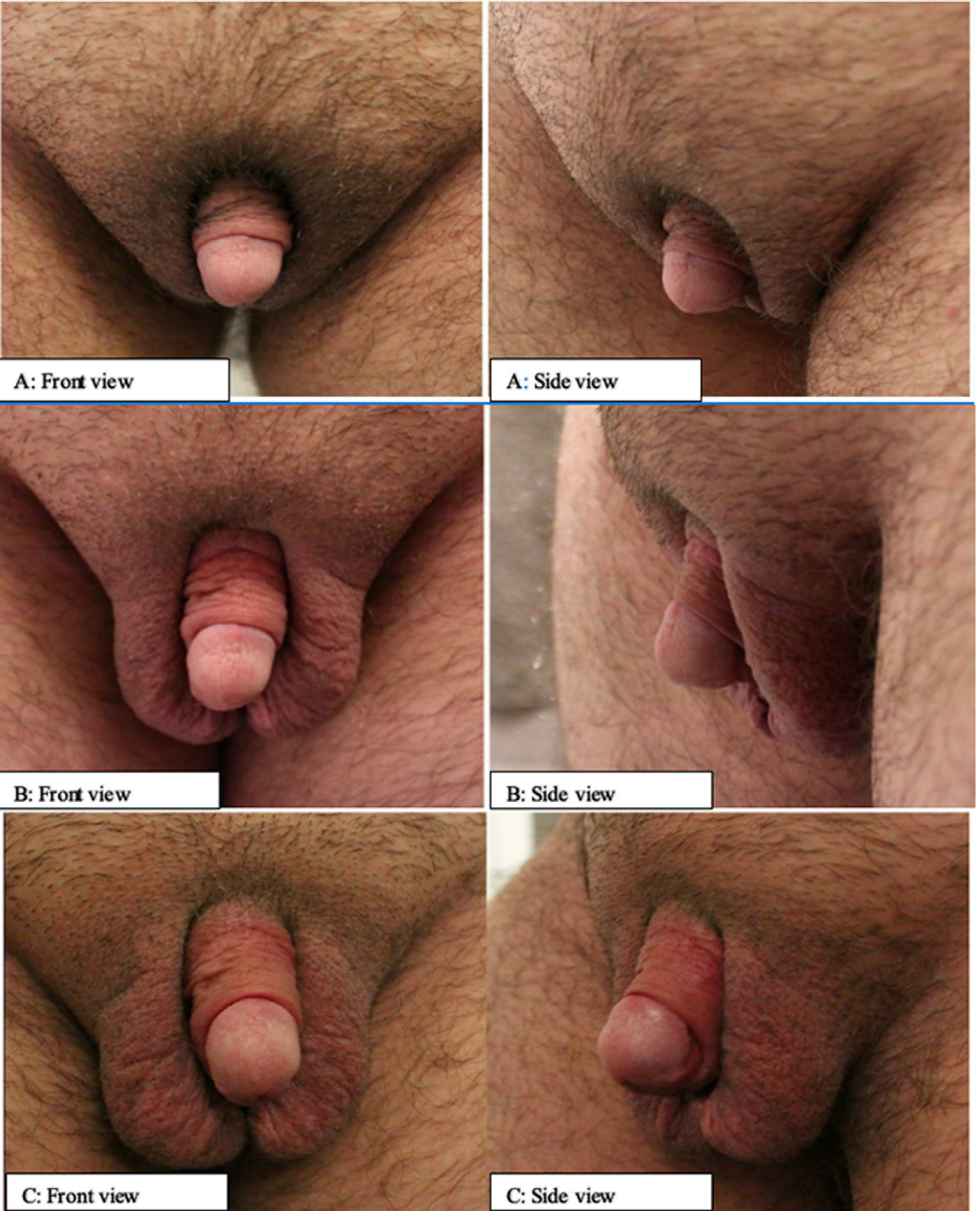
The neophallus of a 29- year-old transman 1 year after metoidioplasty; (A) phallus flaccid, (B) phallus with partial erection without pharmacological intervention, (C) phallus with erection promoted by 20 mg of Tadalafil administered orally 4 hours prior to the photo being taken.

There are currently few studies addressing erectile function, orgasmic function, and the ability to have penetrative intercourse in metoidioplasty patients. Further, no prior studies have reviewed patient attitudes toward erectile aids following metoidioplasty. The specific impact in tumescence, length enhancement, orgasmic dysfunction, or facilitation of penetrative intercourse has not been previously reviewed in this population. Anecdotally, many of our metoidioplasty patients experiment with PDE5i with favorable response (Figure 1). Therefore, we undertook a survey study to investigate the sexual function of patients who have completed metoidioplasty and their experiences using erectile agents. Our primary goal was to first profile the priorities of metoidioplasty patients and rank their perceived importance towards erectile function, orgasmic function and penetrative intercourse. The secondary goals were: (i) evaluate the extent of post-metoidioplasty sexual dysfunction with respect to erection, orgasm and penetration, and (ii) explore whether participants have or would be willing to try PDE5i and ICI as potential erectile aids.

## METHODS

### Study Inclusion & Exclusion Criteria

Patients were included in this study if (i) they were older than 18 years, (ii) their sex assigned female at birth, (iii) they had undergone metoidioplasty surgery, and (iv) they were able to provide informed consent. Individuals were excluded if they failed to meet any of the inclusion criterion or declined invitation to participate in the study.

All adult patients (>18 years old) assigned female at birth, who had undergone metoidioplasty at the Gender Surgery Program of British Columbia (GSP-BC) in Vancouver, British Columbia were invited to participate in the study. Patients were contacted by email or telephone interview in April 2021. The online survey containing the informed consent form was distributed to those who showed interest in the study and made active until the end of June 2021. The Institutional Behavioral Research Ethics Board (BREB) of the University of British Columbia approved of this study prior to distribution of the survey material (BREB number: H20-03782).

### Survey Instrument

The electronic survey was created using Qualtrics software (Qualtrics version 05062021, Provo, UT, USA). The survey consisted of original and self-constructed questions in addition to the validated erection hardness scale (EHS).^14^ We inquired about standard demographic information including age, gender identity, ethnicity, and level of formal education. Additional details including patients’ past and current medical history, mental health diagnoses, medications used, and risk factors for ED (weight, height, smoking status, and alcohol consumption) were collected. The type of metoidioplasty surgery and the date of the procedure was also captured. Participants ranked the importance of the following five parameters on a 5-point Likert scale from “extremely important” to “not at all important”: (i) ability to have penetrative intercourse, (ii) genital sensitivity to touch, (iii) the aesthetic appearance of the neophallus, (iv) ability to achieve and maintain erections, and (v) the ability to orgasm. Participants were specifically asked about their challenges with erectile function, penetrative intercourse, and orgasm and whether they were interested in improving them. Lastly, we asked participants about their past use of and interest in PDE5i and ICI. This included their barriers to access, goals of use, and willingness to try the medications. The full survey is accessible as supplemental material (S1).

### Statistical Analysis

Statistical analyses were performed using Microsoft Excel (version 16.49), Prism-GraphPad (Graphpad software Inc., 2020), and Jamovi (version 1.6.23). Statistical significance was set at a alpha value less than 0.05. Results were reported as median and interquartile range for age, BMI, and time since surgery. For categorical variables, frequency tables were generated. Numerical data were analyzed for measures of central tendency. *T*-test was used to analyze the significance of challenges with erection, orgasm and penetrative intercourse. One-way ANOVA (Welch's test) was utilized to compare the importance of the five sexual function parameters.

## RESULTS

Twenty-two metoidioplasty patients were invited to participate in the study via email. Nineteen patients (86%) indicated interest to participate and received the full survey. Fifteen out of 19 completed the survey, resulting in a completion rate of 79%.

### Participant Demographics

Table 1 summarizes the general and demographic characteristics of the participants. The median age of the participants was 32 -years-old. Median Body Mass Index (BMI) of patients was 25.5 kg/m^2^ ,which falls in the overweight category. The median time since the participants’ metoidioplasty operation to participation in the survey was 15 months. Of the participants, 67% (10/15) self-identified as both male and transmasculine while 33% (5/15) self-identified as only male. One patient identified as male, transmasculine and intersex. Moreover, 67% (10/15) described their ethnicity as Caucasian, 20% (3/15) as mixed, 7% (1/15) as Chinese, and 7% (1/15) did not report their ethnicity.

**Table 1:** Demographics and general characteristics of the study participants

Age (Median years (range))	31.5 (24–57)
BMI (Median kg/m^2^, (range))	25.5 (19.5–37.4)
Ethnicity, n, (%)	
Caucasian/white	10 (67%)
Chinese	1 (7%)
Mixed	3 (20%)
Missing	1 (7%)
Metoidioplasty type*, n (%)	
Full	11 (73%)
Simple	4 (27%)
Time since surgery (median months (range))	15 (6–67)
Smoking Status, n (%)	
Never smoked	10, (67%)
Ex-smoker	5, (33%)
Alcohol Use, n (%)	
Non-drinker	7 (47%)
1-5 glasses/week	5 (33%)
6-10 glasses/week	2 (13%)
11-15 glasses/week	1 (7%)
Education, n (%)	
High school diploma	5 (33%)
Bachelor's degree	8 (53%)
Master's degree	1 (7%)
Doctorate/Ph.D.	1 (7%)

⁎ Full metoidioplasty refers to urethral lengthening in addition to simple metoidioplasty.

Majority of the participants (73%, (11/15)) reported to have been diagnosed with at least one mental health disorder. Of those, 53% (8/15) reported depression, 33% (5/15) reported anxiety, 13% (2/15) reported bipolar disorder and 13% (2/15) reported post-traumatic stress disorder. With regards to smoking, 67% (10/15) of the participants reported to have never smoked, while 33% (5/10) were ex-smokers. 47% (7/15) of the participants don't use alcohol, 33% (5/15) drink 1-5 glasses of alcoholic beverages per week, 13% (2/15) drink 6-10 glasses, and 7% (1/15) drink 11-15 glasses per week (Table 1).

### Sexual Function/Relevant Parameters

**Figure 2:**
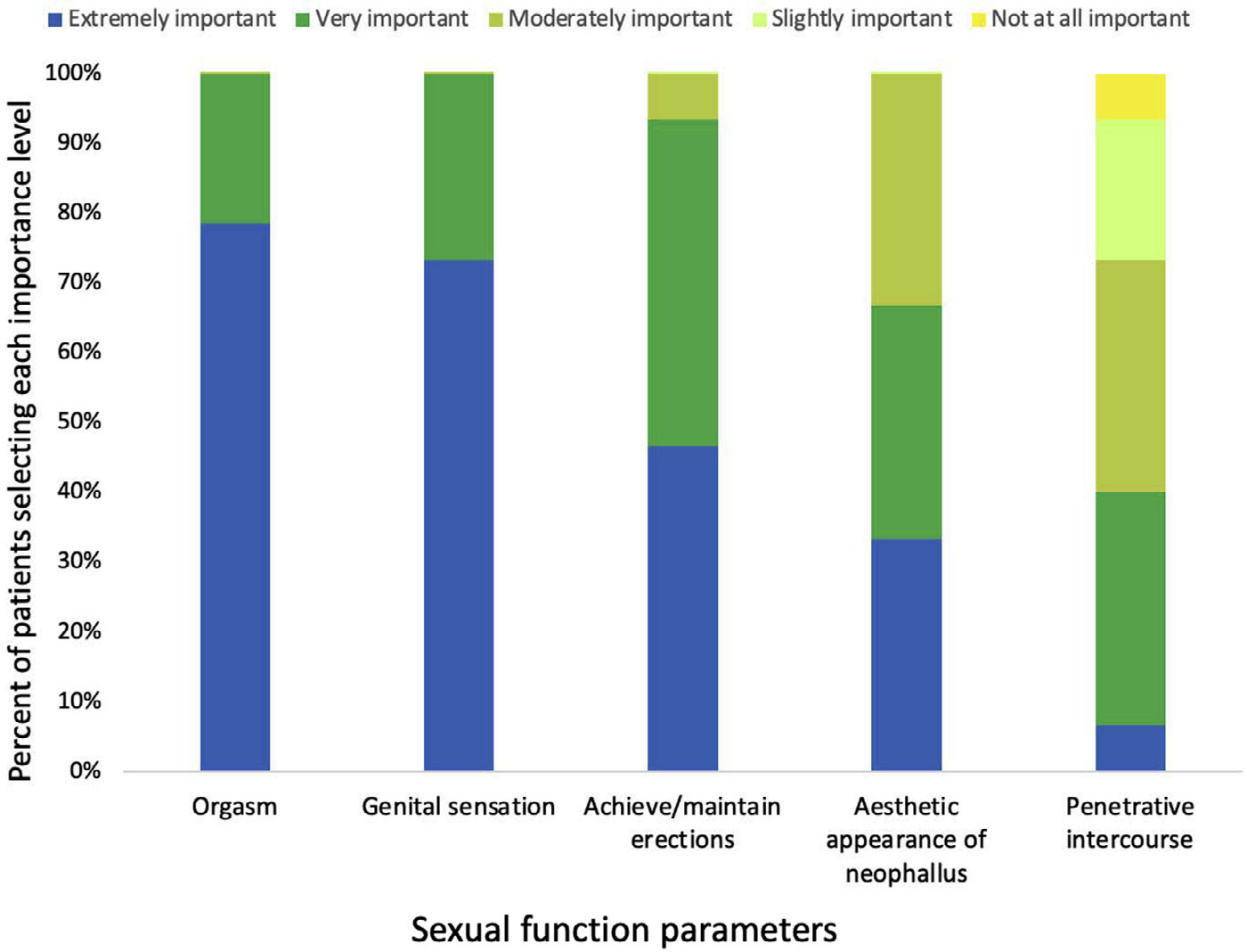
The importance of five sexual parameters among metoidioplasty participants. Fifteen (N = 15) survey participants ranked the importance of each parameter from extremely important to not at all important. The ability to orgasm, genital sensation, and erection were all statistically more important to this population than penetrative intercourse (*P* <.05). No other two parameters were significantly different from each other.

Figure 2 summarizes the participants’ response to the importance of the five sexual relevant parameters assessed in this study after metoidioplasty. All participants ranked the ability to orgasm and preservation of genital sensation to touch and pressure as either extremely important or very important to them.

The ability to achieve and/or maintain erections was ranked extremely important or very important by 93% of participants (14/15). The aesthetic appearance of phallus was ranked as extremely important or very important by 67% (10/15) of patients. Lastly, 40% (6/15) ranked the ability to have penetrative intercourse extremely important or very important (Figure 2). Penetrative intercourse was significantly less important to the participants than orgasm, genital sensation, and erectile function (all *P* <.05).

### Sexual Function Challenges

**Figure 3:**
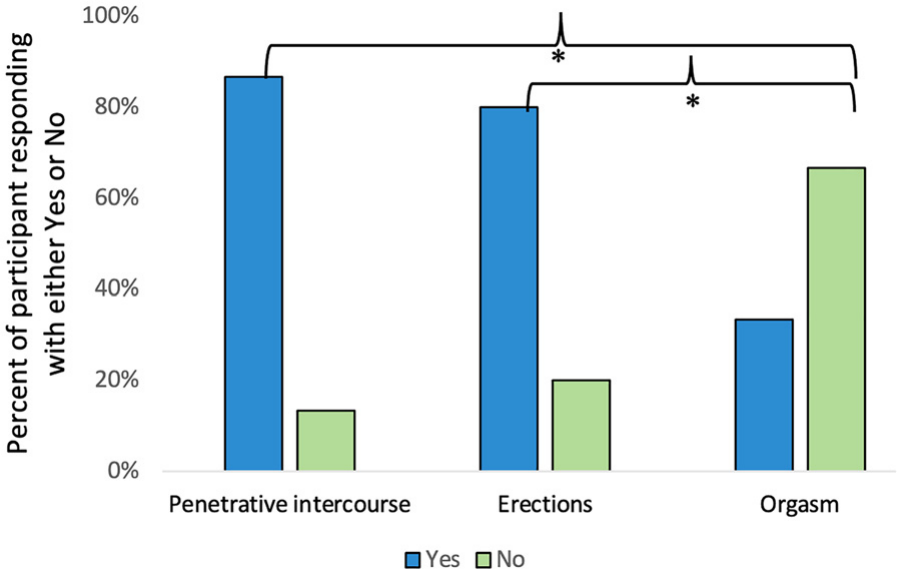
Participants’ response to whether they were experiencing challenges with penetrative intercourse, erections, and the ability to orgasm. Survey participants (N = 15) responded with “yes” or “no” but also had the option to provide more explanation. The “yes” response to challenges with penetrative intercourse and erections was significantly higher than orgasm (*P* <.05) as denoted by the asterisk. Penetrative intercourse and erections were not statistically different (*P* =.88).

The prevalence of sexual challenges such as erectile dysfunction, and difficulty with orgasm and penetrative intercourse after metoidioplasty was assessed. Of the participants, 87% (13/15) reported to be challenged in performing penetrative intercourse as the insertive partner. Moreover, 80% (12/15) of the patients also faced challenges with their ability to achieve or maintain erections. In contrast, only 33% (5/15) of the participants experienced challenges in achieving orgasm (Figure 3). There were no statistical differences between the proportion of the participants bothered by problems with erectile function and penetrative intercourse (*P* =.88). However, a significantly larger proportion of the participants were experiencing challenges with penetrative intercourse and erectile function compared to those experiencing challenges with orgasm (*P* <.05).

### Erection Hardness Score

Participants were asked to describe their natural erections, without the use of any pharmacotherapy based on the EHS questionnaire, with multiple selections allowed. Of the 14 participants who recorded a response to the EHS questionnaire, 4 participants selected 2 options, while the remaining 10 selected only one option. One participant did not respond to this question. This resulted in 18 responses in total.

**Figure 4:**
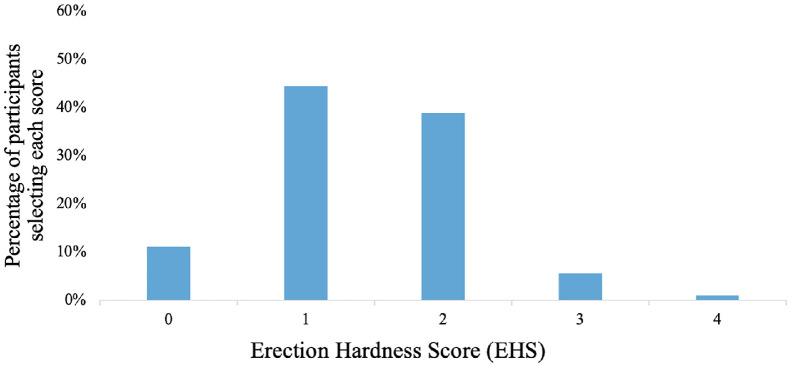
Participants response to the Erection Hardness Score (EHS) questionnaire. Numbers listed on the horizontal axis depict the following: 0 =“penis does not enlarge,” 1 =“penis is larger but not hard,” 2 = “penis is hard but not hard enough for penetration,” 3 = “penis is hard enough for penetration but not completely hard,” 4 = “penis is completely hard and fully rigid.” Note: participants were allowed to choose more than one option. Fourteen participants recorded 18 responses (N = 18).

The survey showed that 44% of the participants described their erections as “larger but not hard.” Furthermore, 39% ranked their erections as “hard but not hard enough for penetration” (Figure 4). Only 6% reported that their erections are “hard enough for penetration but not completely hard.” Lastly, 11% reported that their “penis does not enlarge.” The proportion of participants who chose a score of 1 or 2 was statistically higher than those who selected other scores, *X*^2^ (4, *N* = 18) = 14.8, *P* =.005. This indicates overall a significant failure to achieve full erections.

### PDE5 Inhibitors

Of the participants, 53% (8/15) had never used PDE5i, 13% (2/15) had used them once, and 33% (5/15) had used them more than once. All participants, who had tried these medications, had initially received them as samples from a physician, and three participants also received a prescription. Of those participants who had used PDE5i, 86% (6/7) did so to achieve or improve their erections. Furthermore, 71% (5/7) also aimed to improve their ability to have penetrative intercourse. 43% (3/7) used PDE5i with an additional goal of improving their orgasms. Lastly, one participant had solely used the medication to improve blood flow and promote better healing after the surgery, as suggested by their surgeon. With regards to overall satisfaction, 43% (3/7) of participants were extremely satisfied with the results of PDE5i, while 57% (4/7) were somewhat satisfied.

Of all participants, 87% (13/15) were willing to try PDE5i in the future. Seven percent (1/14) were not willing to use these medications, as they found them ineffective due to the size of their phallus. Lastly, 7% (1/14) were unsure about their willingness to try such drugs due to adverse effects they had experienced with the medication in the past.

Participants further ranked their goals for using PDE5i in the future. 93% (13/14) reported achieving erections with PDE5i drugs as either extremely important or very important to them. Seventy-one percent (10/14) ranked orgasm as extremely important or very important. Furthermore, 57% ranked the goal of penetrative intercourse with the same importance.

### Intracavernosal Injections

No participants had previously used ICI. A total of 40% (6/15) of the participants were willing to try this treatment, 33% (5/15) were unsure, and 27% (4/15) were unwilling to use them. The reason for unwillingness to try were invasiveness, fear of self-injection, and the belief that even with the injection, their phallus would be too small for penetrative sex.

Participants further ranked their goals for use of ICI in the future. A total of 92% (12/13) of the participants reported improving erections as either extremely important or very important with the use of ICI. 62% (8/13) ranked their goal to improve their orgasms as extremely important or very important. Lastly, 54% ranked their goals for penetrative intercourse with the same importance.

## DISCUSSION

In this study, several parameters of sexual function post-metoidioplasty were explored. For our post-metoidioplasty patients, the ability to orgasm, preservation of erogenous genital sensation, and the ability to achieve or maintain erections were extremely important. Since metoidioplasty outcomes are not focused on the development of a large externally virilized phallus, it is common to devalue the role of erectile function in sexual satisfaction of this patient population. In fact, no prior publications have addressed the importance of the ability to achieve or maintain erections post-metoidioplasty. The observation that 93% of the participants ranked this parameter as extremely or very important illustrates the significance of this finding.

This also suggests a shift in the priorities of the TM patients before versus after genital gender affirming surgery. In a sample of 336 TM patients, who had not had surgery and were receiving intramuscular androgen injections to treat gender dysphoria, the primary goals of hormonal treatment were cessation of menses (52.7%) and achieving a deeper voice (32.4%).^15^ Notably, participants were permitted to select a single dominant reason for seeking hormone therapy out of nine choices that included clitoral growth and increased libido. None of the TM chose either of those sex parameters as the main reason for seeking gender-affirming hormone treatment.

In Table 1, we present a variety of demographic and health-related variables that may relate to erectile function. This includes mental health, smoking, alcohol consumption, and BMI.^15^, ^16^, ^17^ Unfortunately, our sample size is too small to correlate these variables with the primary results we have for the metoidioplasty population facing sexual challenges and their willingness to pursue remedial measures.

Compared to phalloplasty, metoidioplasty patients have reported a statistically higher satisfaction with the sexual functioning of their neophallus after surgery. The authors suggested that this may be due to increased erogenous sensation following metoidioplasty compared to phalloplasty, and patients’ acceptance that they are unlikely to acquire a phallus large enough for penetrative intercourse.^18^ This finding further supports that erogenous sensation, the ability to orgasm, and erectile function appear to be more important than the ability to have penetrative intercourse for patients who have undergone metoidioplasty. These results can facilitate decision-making for patients who are exploring options in regard to bottom gender-affirmation surgery.

We observed that most participants faced challenges with the ability to have penetrative intercourse and to achieve or maintain an erection. This finding is consistent with previous studies suggesting that most metoidioplasty patients lacked the ability to achieve penetration.^19^ In contrast, only a minority of our participants experienced difficulties with orgasm. Several studies support that the ability to orgasm is commonly preserved after metoidioplasty, further validating our results.^20^^,^^21^

In a prior study, metoidioplasty patients reported moderate growth of the phallus with an average of 1.7 cm during arousal.^22^ This finding is consistent with our results in that the ability to have an erection is achievable post-metoidioplasty. We have further characterized this ability, showing that the majority of patients would be interested in improving their erections from a EHS score of 1 or 2 (ie, “penis is larger but not hard” or “penis is hard but not hard enough for penetration,” respectively) to a higher EHS score.

The use of PDE5i and ICI as erectile aids in the TM populations has not been previously studied. This survey demonstrates that the majority of metoidioplasty patients are willing to try PDE5is. However, participants reported that a “lack of knowledge and understanding from primary care physicians” were a major barrier to access this treatment. Furthermore, individuals noted the current scarcity of data to understand which medication would best suit their needs, was another barrier in their willingness to try the therapies.

Our findings demonstrate that the most important goal for patients, who wished to use of PDE5i, was to improve their erection, but not solely for the purpose of penetrative intercourse. Participants also highly rated the goal of using PDE5is as improving their orgasms. Similar goals were reported in considering ICIs. However, participants were less inspired to try ICIs due to fear of self-injection and discomfort in seeking such a treatment from their primary care physicians.

Our results suggest that the significance of penile erections in term of perceived masculinity may be somewhat different for TM individuals than for cismen. There is extensive academic literature on the “coital imperative” that comes out of the feminist critique of phallocentrism.^23^^,^^24^ The “coital imperative” implies that to be the insertive partner in sex matters most to cismen. While this idea has been challenged by various feminist authors, data on the impact of ED from prostate cancer treatments on men's self-esteem and sense of their manhood fit the model.^25^^,^^26^ Indeed, the gold standard for measuring erectile function, the International Index of Erectile Function,^27^ is built on the idea that what matters most to the heterosexual natal male is having erections firm enough for penetration. Although our sample is small, our data suggest that TM, who had received a metoidioplasty, do not necessarily see penetrative sex as definitional to their manhood. In simple terms, the significance of penile erections may differ for men depending on whether they have never had penile-insertive sex versus having had it in the past but lost it due to disease or treatment.

Transmen and gender diverse individuals who were assigned female at birth may also self-identify as gender non-binary, gender non-conforming, genderqueer, agender, intersex, or Two-Spirit persons. While most of the participants in our study self-identified as male and transmasculine, the results could be broadened to other individuals who are considering lower gender-affirming surgery.

### Strengths and Limitations

This study represents the first assessment of erectile aid use in the TM population post-metoidioplasty. Although this study has a relatively small sample size, this represents one of the largest cohorts of metoidioplasty patients in North America. All the enrolled patients had undergone the surgery in the same Centre, so the outcomes of the surgery might have been affected by the same procedural imperfections. This bias could be minimized by a future multicenter study design. This survey study is also limited by the inherent inability to verify clinical data reported via online surveys. In addition, online survey distribution to patients introduces a certain degree of recall and selection bias. The majority of survey questions were self-constructed and non-validated in this population. Lastly, a lower than anticipated completed survey responses limited the statistical power of the analysis.

## CONCLUSIONS

The vast majority of participants in our study described the ability to achieve and/or maintain erection as extremely important or very important. Unfortunately, most participants indicated difficulty in achieving firm erections without treatment and no participants reported having achieved a fully rigid erection. Most were willing to try PDE5i to achieve a better erection, whereas fewer participants showed interest in trying ICI. Participants commonly reported that a “lack of knowledge and understanding from primary care physicians” were major barriers to access this treatment. Future research is warranted on evaluation of the efficacy of PDE5i and ICI as potential treatments of ED in TM population after metoidioplasty.

## ETHICAL STATEMENT

The authors are accountable for all aspects of the work in ensuring that questions related to the accuracy or integrity of any part of the work are appropriately investigated and resolved.

## STATEMENT OF AUTHORSHIP

Conceptualization, A.K1., E.B., R.W., and A.K10.; Methodology, A.K1., and E.B; Investigation, A.K1., E.B., S.K., and A.K10.; Writing – Original Draft, A.K1. and R.W.; Writing – Review & Editing, S.K., E.B., C.O., S.M., L.W., and P.M..; Funding Acquisition, A.K10. and E.B.; Supervision, R.W., K.G., and A.K10.

## Appendix Supplementary materials

Image, application 1

## Footnotes

Supplementary material associated with this article can be found in the online version at doi:10.1016/j.esxm.2022.100505.
