# Supplementary material for: The Sexual Goals of Metoidioplasty Patients and Their Attitudes Toward Using PDE5 Inhibitors and Intracavernosal Injections as Erectile Aids
Source: Sex Med. 2022 Apr 8;10(3):100505. doi: 10.1016/j.esxm.2022.100505 (PMC9177887; doi:10.1016/j.esxm.2022.100505)
Supplement: Supplementary file 1 [file mmc1.docx]

**Start of Block: Personal information**

Q1 What is your age?

________________________________________________________________

Q2 What is your current gender identity? (feel free to choose as many as you want from the following list or add one in the "other" option)

- Male (1)
- Female (2)
- Trans masculine (3)
- Trans feminine (4)
- Non-binary (5)
- Agender (6)
- Two-spirit (7)
- Inter-sex (8)
- Gender fluid (9)
- Other: (10) ________________________________________________

Q3 What is your highest level of formal education?

- No formal education (1)
- Middle school (2)
- High school (or equivalent) (3)
- Bachelor's degree (4)
- Master's degree (5)
- Doctorate/PHD (6)

Q4 How would you describe your ethnicity?

________________________________________________________________

**End of Block: Personal information**

**Start of Block: Health and risk factors**

Q5 What is your height in centimeters?

________________________________________________________________

Q6 What is your current weight in kilograms?

________________________________________________________________

Q7 Do you have or have you ever been diagnosed with any of the following conditions? (please select all that apply)

- Diabetes (1)
- Hypertension (high blood pressure) (2)
- Peripheral vascular disease or injury (3)
- High blood cholesterol (4)
- Depression (5)
- Chronic sleep disorders (including obstructive sleep apnea) (6)
- Other mental illnesses (please specify) (7) ________________________________________________
- Other (please specify) (8) ________________________________________________

Q8 Which of the following is closest to your current smoking/chewing tobacco status? (select all that apply)

- Never used tobacco (1)
- Current smoker (2)
- Ex-smoker (3)
- Current tobacco chewer (4)
- Ex-tobbaco chewer (5)

Q9 How often do you drink alcohol?

- Never (1)
- 1-5 glasses per week (2)
- 6-10 glasses per week (3)
- 11-15 glasses per week (4)
- More than 15 glasses per week (5)

Q10 What medications (prescribed or over the counter) or supplements do you take regularly?

________________________________________________________________

________________________________________________________________

________________________________________________________________

________________________________________________________________

________________________________________________________________

**End of Block: Health and risk factors**

**Start of Block: Metoidioplasty**

Q11 Have you had a metoidioplasty surgery?

- Yes (1)
- No (2)

*Skip To: End of Block If Have you had a metoidioplasty surgery? = No*

Q12 What type of metoidioplasty procedure did you have?

- Simple release (simple meta without urethral lengthening) (1)
- Full metoidioplasty (simple meta + urethral lengthening) (2)
- Other: please explain (3) ________________________________________________

Q13 When was your metoidioplasty procedure done? (MM/YYYY)

________________________________________________________________

**End of Block: Metoidioplasty**

**Start of Block: Sexual aspects of erectile aids**

Q14 How important are the following options to you (with respect to your genitals)? 
Please rank each of these on a scale from not at all important to extremely important.

|  | Extremely important (1) | Very important (2) | Moderately important (3) | Slightly important (4) | Not at all important (5) |
| --- | --- | --- | --- | --- | --- |
| Ability to have penetrative intercourse (as the insertive partner) (1) |  |  |  |  |  |
| Genital sensitivity to touch and/or pressure (2) |  |  |  |  |  |
| Aesthetic appearance (3) |  |  |  |  |  |
| Ability to achieve and maintain an erection (4) |  |  |  |  |  |
| Ability to achieve an orgasm (5) |  |  |  |  |  |

Q15 Are you currently interested in achieving or improving the ability to have penetrative intercourse (as the insertive partner)

- Yes (1)
- No (2)

Q16 Are you currently interested in improving the ability to achieve or maintain erections?

- Yes (1)
- No (2)

Q17 Are you currently interested in achieving or improving orgasms?

- Yes (1)
- No (2)

Q18 Are you currently facing challenges with penetrative intercourse (as the insertive partner)?

- Yes (1)
- No (2)

Q19 Are you currently facing challenges with your ability to achieve or maintain an erection?

- Yes (1)
- No (2)

Q20 Are you currently facing challenges in your ability to orgasm?

- Yes (1)
- No (2)

Q21 Which of the following best describes the erections you have most often? (we do not know what you call your genitals so we apologize if you do not self-identify with the term penis)

- Penis does not enlarge (1)
- Penis is larger, but not hard (2)
- Penis is hard, but not hard enough for penetration (3)
- Penis is hard enough for penetration but not completely hard (4)
- Penis is completely hard and fully rigid (5)
- Other (please explain) (6) ________________________________________________

**End of Block: Sexual aspects of erectile aids**

**Start of Block: Erectile aids: PDE5Is**

Q22
**What are "PDE5 inhibitors"?**
 PDE5 inhibitors are oral drugs (pills) mainly used to treat erectile dysfunction in cismen. Some are also used to treat subtypes of high blood pressure in the lungs and prostate enlargement. Four major commonly known PDE5 inhibitors are Viagra, Cialis, Levitra and Stendra. 


These medications work by making the blood vessels in the area larger, which allows more blood to fill the tissues. Within 30-60 minutes,  the penis/clitoris will swell and harden and become more sensitive to touch. 


Although PDE5 inhibitors are generally well tolerated, they can have side effects such as headaches, nausea, dizziness, flushing, indigestion, nasal congestion and rhinitis.

Q23 Have you ever used PDE5 inhibitors?

- No (1)
- Yes, once (2)
- Yes, more than once (4)
- Unsure (3)
- Other: (5) ________________________________________________

*Skip To: Q27 If Have you ever used PDE5 inhibitors? = No*

*Skip To: Q27 If Have you ever used PDE5 inhibitors? = Unsure*

Q24 Did you obtain the medication from a physician or a healthcare provider?

- Yes, as a sample (1)
- Yes, as a prescription (2)
- No (please explain) (3) ________________________________________________

Q25 What goals were you trying to achieve by using PDE5 inhibitors? (choose all that apply)

- Erections (To have or improve) (1)
- Orgasms (To have or improve) (2)
- Penetrative intercourse (To have or improve) (3)
- Other sex-related goals (please explain) (4) ________________________________________________
- Non-sex-related goals (please explain) (5) ________________________________________________

Q26 How satisfied were you with the results of PDE5 inhibitors?

- Extremely satisfied (ie. it had a very positive effect) (1)
- Somewhat satisfied (ie. it had a positive effect) (2)
- Neither satisfied nor dissatisfied (3)
- Somewhat dissatisfied (ie. it had no effects) (4)
- Extremely dissatisfied (ie. there were negative effects) (5)
- Other (please explain) (6) ________________________________________________

Q27 Would you be willing to use PDE5 inhibitors in the future?

- Yes (1)
- No (please explain) (2) ________________________________________________
- Unsure (3)
- Other (please explain) (4) ________________________________________________

*Skip To: Q29 If Would you be willing to use PDE5 inhibitors in the future? = No (please explain)*

Q28 What will be your goals when you try PDE5 inhibitors in the future? (please rank each of these from not at all important to extremely important)

|  | Extremely important (1) | Very important (2) | Moderately important (3) | Slightly important (4) | Not at all important (5) |
| --- | --- | --- | --- | --- | --- |
| Erections (to have or improve) (1) |  |  |  |  |  |
| Orgasms (to have or improve) (2) |  |  |  |  |  |
| penetrative intercourse (to have or improve) (3) |  |  |  |  |  |
| Other sex-related goal(s): please explain (4) |  |  |  |  |  |
| Non-sex-related goal(s): please explain (5) |  |  |  |  |  |

Q29 What barriers do you think you might face in accessing or using PDE5 inhibitors?

- Accessing (getting a prescription, etc.) (1) ________________________________________________
- Using (taking the pills, side effects, etc.) (2) ________________________________________________

Q30 Can you think of anything else that we have not asked about PDE5 inhibitors?

________________________________________________________________

________________________________________________________________

________________________________________________________________

________________________________________________________________

________________________________________________________________

**End of Block: Erectile aids: PDE5Is**

**Start of Block: Erectile aid: ICI**

Q31
What are "intracavernosal injections"? 

Intracavernosal injections (ICIs) are medications injected into the base of the penis or clitoris. These are typically used to check for or treat erectile dysfunction in cismen. 


These medications work by making the blood vessels in the area larger, which allows more blood to fill the tissues. Within 10-15 minutes,  the penis/clitoris will swell and harden and become more sensitive to touch. 


Possible side effects of ICIs are prolonged erections, penile pain, penile fibrosis, occurrence of hematoma, and formation of nodules.

Q32 Have you ever used intracavernosal injections?

- No (10)
- Yes, once (11)
- Yes, more than once (12)
- Unsure (please explain) (13) ________________________________________________
- Other (please explain) (14) ________________________________________________

*Skip To: Q36 If Have you ever used intracavernosal injections? = No*

Q33 Did you obtain the medication from a physician or a healthcare provider?

- Yes, as a sample (1)
- Yes, as a prescription (2)
- No (please explain) (3) ________________________________________________

Q34 What goals were you trying to achieve by using ICIs? (choose all that apply)

- Erections (to have or improve) (1)
- Orgasms (to have or improve) (2)
- Penetrative intercourse (to have or improve) (3)
- Other sex-related goal(s) (please explain) (4) ________________________________________________
- Non-sexual goal(s) (please explain) (5) ________________________________________________

Q35 How satisfied were you with the results of ICIs?

- Extremely satisfied (ie. it had a very positive effect) (1)
- Somewhat satisfied (ie. it had a positive effect) (2)
- Neither satisfied nor dissatisfied (3)
- Somewhat dissatisfied (ie. it had no effects) (4)
- Extremely dissatisfied (ie. there were negative effects) (5)
- Other (please explain) (6) ________________________________________________

Q36 Would you be willing to use ICIs in the future?

- Yes (1)
- No (please explain) (2) ________________________________________________
- Unsure (3)
- Other (please explain) (4) ________________________________________________

*Skip To: Q38 If Would you be willing to use ICIs in the future? = No (please explain)*

Q37 What will be your goals when you try ICIs in the future? (please rank each of these from not at all important to extremely important)

|  | Extremely important (1) | Very important (2) | Moderately important (3) | Slightly important (4) | Not at all important (5) |
| --- | --- | --- | --- | --- | --- |
| Erections (to have or improve) (1) |  |  |  |  |  |
| Orgasms (to have or improve) (2) |  |  |  |  |  |
| Penetrative intercourse (to have or improve) (3) |  |  |  |  |  |
| Other sex-related goal(s): please explain (4) |  |  |  |  |  |
| Non-sex-related goal(s): please explain (5) |  |  |  |  |  |

Q38 What barriers do you think you might face in accessing or using ICIs?

- Accessing (getting a prescription, etc.) (1) ________________________________________________
- Using (taking the pills, side effects, etc.) (2) ________________________________________________

Q39 Can you think of anything else that we have not asked about intracavernosal injections?

________________________________________________________________

________________________________________________________________

**End of Block: Erectile aid: ICI**

**Start of Block: Comments and feedback**

**You have now reached the end of the survey questions.**
**Feel free to give us feedback on the survey below if you want, or click onto the next page to end the survey.**

Q40 Please let us know if you felt that any wording or questions in the survey was not inclusive, confusing or incorrect

________________________________________________________________

________________________________________________________________

________________________________________________________________

________________________________________________________________

________________________________________________________________

Q41 We appreciate any other comments or feedback you might have regarding this survey.

________________________________________________________________

________________________________________________________________

**End of Block: Comments and feedback**
